# The Effect of Amlodipine Alone and in Combination with Atenolol on Bowel Habit in Patients with Hypertension: An Observation

**DOI:** 10.5402/2011/757141

**Published:** 2010-11-07

**Authors:** Lekha Saha, Chander Shekhar Gautam

**Affiliations:** ^1^Department of Pharmacology, Postgraduate Institute of Medical Education and Research (PGIMER), Chandigarh 160012, India; ^2^Department of Pharmacology, Government Medical College and Hospital, Chandigarh 160032, India

## Abstract

The prevalence of hypertension increases with advancing age. The management of hypertension especially in the elderly has its own limitations. Verapamil is not recommended in the elderly on account of high incidences of troublesome constipation. Amlodipine has become very popular with the cardiologists and general physicians. Survey of literature has not yielded any citation where the troublesome effect of amlodipine on the gastrointestinal tract has been reported. In an experimental study on isolated rabbit intestine we have demonstrated that amlodipine dose-dependently inhibit the spontaneous activity of the intestinal tract. With this background the present observational study was planned. A total of 100 hypertensive patients were included in the present study. Fifty patients were on amlodipine alone and 50 patients on combination of amlodipine and atenolol. The main parameter analyzed was the frequency and consistency of stool before and after intake of drug. The relative risk (RR) of developing constipation was 4.00 with 95% CI 0.8930 to 17.917 in amlodipine alone group. From this study it can be concluded that the relative risk of developing constipation is 4 times more in patients who are taking amlodipine alone as compared to those patients who are on combination of amlodipine and atenolol.

## 1. Introduction

The prevalence of hypertension increases with advancing age; for example, about 50% of people between the ages of 60 and 69 years old have hypertension, and the prevalence is further increased beyond age 70 [1]. The management of hypertension especially in the elderly has its own limitations. Drugs used in hypertension are calcium channel blockers (CCBs), angiotensin converting enzyme inhibitors (ACEIs), and low-dose diuretics [2]. Verapamil, belonging to papaverine group, is not recommended in the elderly on account of high incidences of troublesome constipation [2]. Amlodipine, pharmacokinetically the most distinct type of dihydropyridine (DHP) CCB, has become very popular with the cardiologists and general physicians in doses of 5 mg or 10 mg once daily [3, 4]. Survey of the literature has not yielded any citation where the troublesome effect of amlodipine on the gastrointestinal tract (GIT) has been reported [5]. We had demonstrated in an experimental study on isolated rabbit intestine that amlodipine dose dependently inhibits the spontaneous activity of the intestinal tract [6]. With this background the present observational study was planned to demonstrate the experimental observation in the hypertensive patients' population coming to the medical OPD in our hospital. Therefore, the aim of the present study was to demonstrate the effect of amlodipine alone and in combination with atenolol on the bowel habits in patients who are taking these drugs.

## 2. Materials and Methods

This was a noninterventional observational study. The present study was approved by local institutional ethics committee. The subject of the present study was the patient population attending the hypertensive clinic in our institution. Both male and female patients with age 40–70 years, taking amlodipine alone or in combination with atenolol, were screened for this present study. Patients with history of intake of any other medicines or history of any other diseases that can decrease intestinal motility like hypothyroidism were excluded from the study. Written informed consent was taken from the patients before being included in the study. A questionnaire-based proforma was filled for each patient which includes the frequency and consistency of the stool before and after the drug intake, presence of blood in stool and hard stool, the duration of drug intake, the dose of the drug, the use of laxatives, physical activity, and diet, and was filled for each patient. The answers were then compiled, tabulated, and analyzed. The constipation was defined as the history of less than 3 spontaneous bowel movements (SBM) per week for a period of at least 6 months [7].

## 3. Statistical Analysis

The data are expressed as mean ± SD and as absolute number of patients whenever applicable. The relative risk of developing constipation and blood in stool and hard stool was calculated in both the patient groups who are taking amlodipine alone or in combination with atenolol by using Fisher's exact test. The other variables like age, sex, SBM/wk, and duration of treatment were compared by using unpaired “*t*” test. *P* < .05 was considered statistically significant.

## 4. Results

A total of 100 patients who attended the hypertensive clinic in Government Medical College and Hospital, Chandigarh, were included in the study after the screening. Fifty patients were on amlodipine alone and 50 patients were on combination of amlodipine and atenolol.

Table 1 depicted the data of all 100 patients. There was no significant difference in the baseline characteristics in both the groups. The baseline SBM/wk was comparable (8.64 ± 4.4 versus 7.84 ± 2.5) in both the groups (Table 1). The number of patients in the amlodipine group with SBM/wk less than 3 was 8 whereas in combination group (amlodipine + atenolol) it was 2 (*P* = .045, Fisher's exact test, significant). The relative risk (RR) of developing constipation was 4.00 with 95% CI 0.8930 to 17.917 in amlodipine alone group. However, there was no significant difference in SBM/wk in both the groups before and after treatment (*P* > .05). The majority of the patients in both the groups were on amlodipine 10 mg daily dose (28(56%) patients in amlodipine group and 29(58%) patients in combination group) (Table 1). The duration of treatment with amlodipine was also comparable in both the groups (16.75 ± 2.5 months versus 14.025 ± 2.58 months). The number of patients with blood in stool was 5 in amlodipine group while there was none in the combination group (*P* = .028, significant). Straining/hard stool was seen in 10 patients in the amlodipine group and 3 patients in the combination group (*P* = .035, significant) (Table 1). The risk (RR = 3.33 with 95% CI 0.9748 to11.399) of developing hard stool was more in amlodipine group. The number of patients taking laxatives was 5 in amlodipine alone group and 4 in combination group (*P* > .05).

## 5. Discussion

So from the present observation, it can be said that amlodipine alone associated with increased incidence of constipation (RR = 4.00) and hard stool (RR = 3.333) and when atenolol was combined, the incidence of constipation and hard stool was less (*P* < .05). Like our previous animal study, this observational study also demonstrated that amlodipine can reduce the peristaltic activity of the intestine when used alone. From this observation and previous animal study, we can hypothesized that when amlodipine is used alone it could reduce the peristaltic activity of the intestine which may be due to the release of catecholamines (like nifedipine) [5], and subsequent interaction of this catecholamines with the adrenergic receptors present in the gastrointestinal tract may lead to constipation. However, when amlodipine was combined with atenolol, the released catecholamine was unable to produce an effect as *β*-adrenergic receptor in the GIT was blocked by atenolol. The Indian populations, especially the advancing age group, are very much concerned about their bowel evacuation. Such hypertensive patients who suffer constipation on account of the drug can also have the mental stress which can further facilitate the release of catecholamines and may have deleterious effect on the bowel habits. Hence, in an elderly patient having concomitant compromised GIT and hypertension warrants a judicious use of amlodipine. Further study is in progress.

## Figures and Tables

**Table 1:** Effects of amlodipine alone and in combination with atenolol on bowel habit in patients with hypertension. SBM: spontaneous bowel movement. Results are expressed as mean ± SD and absolute number.

Parameters	Amlodipine (*n* = 50)	Amlodipine + atenolol (*n* = 50)
Age (Yrs) (M ± SD)	54.14 ± 11.32	54.36 ± 10.27
Male : Female	23 : 27	21 : 29
SBM/wk (M ± SD)		
Before treatment	8.64 ± 4.4	7.84 ± 2.5
After treatment	8.0 ± 4.7	7.44 ± 2.64
No of patients with SBM/wk < 3	8*	2
No. of patients with blood in stool	5**	0
No. of patients with straining/hard stool	10***	3
Duration of treatment (Months) (M ± SD)	16.75 ± 2.5	14.025 ± 2.6
Dose of amlodipine		
No. of patients taking 5 mg OD	22	21
No. of patients taking 10 mg OD	28	29
No. of patients taking isabgual	5	4

*P = .045, Fisher's exact test, RR = 4.00.

**P = .028, Fisher's exact test.

***P = .035, Fisher's exact test, RR = 3.33.
